# Service utilization in community health centers in China: a comparison analysis with local hospitals

**DOI:** 10.1186/1472-6963-6-93

**Published:** 2006-08-03

**Authors:** Xilong Pan, Hassan H Dib, Xiaohang Wang, Hong Zhang

**Affiliations:** 1 Department of Health Policy and Management, School of Public Health, Peking University, China

## Abstract

**Background:**

Being an important part of China's Urban Health Care Reform System, Community Health Centers (CHCs) have been established throughout the entire country and are presently undergoing substantial reconstruction. However, the services being delivered by the CHCs are far from reaching their performance targets. In order to assess the role of the CHCs, we examined their performance in six cities located in regions of South-East China. The purpose of this investigation was to identify the utilization and the efficiency of community health resources that are able to provide basic medical and public health services.

**Methods:**

The study was approved by Peking University Health Science Center Institutional Reviewing Board (NO: IRB00001052-T1). Data were collected from all the local health bureaux and processed using SPSS software. Methods of analysis mainly included: descriptive analysis, paired T-test and one-way ANOVA.

**Results:**

The six main functions of the CHCs were not fully exploited and the surveys that were collected on their efficiency and utilization of resources indicate that they have a low level of performance and lack the trust of local communities. Furthermore, the CHCs seriously lack funding support and operate under difficult circumstances, and residents have less positive attitudes towards them.

**Conclusion:**

The community health service must be adjusted according to the requirements of urban medical and health reform, taking into account communities' health needs. More research is required on the living standards and health needs of residents living within the CHC's range, taking into consideration the users' needs in expanding the newly implemented service, and at the same time revising the old service system so as to make the development of CHCs realistic and capable of providing a better service to patients. Several suggestions are put forward for an attainable scheme for developing a community health service.

## Background

Since they are considered an important part of the Chinese Urban Health Reform System, Community Health Centers (CHCs) have been established throughout the entire nation; they are currently undergoing substantial re-construction. Until 2002, 31 provinces including the autonomous regions and central government-ruled cities such as Beijing, Shanghai, ChongQing and Tianjin had a total of around 2406 CHCs and 9700 service stations. However, these CHCs are facing many problems in delivering their services, attributable to the different speeds of development among centers, lack of resources, and imbalance in the sizes of CHCs, so it is difficult for them to meet citizens' needs. Nevertheless, the CHCs are considered the main primary institutions for offering basic medical and public health services. They are regarded as the basic networks for medical treatment and public health surveillance. Therefore, redistributing health resources towards CHCs can ensure social health equality [1].

Traditional health methods such as Traditional Chinese Medicine (TCM) concentrate more on cure than prevention and/or care. By the beginning of the 21^st ^century, traditional methods could no longer cope with the tasks of the new CHCs or patients' health service demands such as care, emergency treatment and rehabilitation. Hence, there is an urgent need to develop the CHC services in order to meet the residents' demands. The main question is how to develop these centers and expand them, and at the same time be able to attract patients to use them. Another important question is how to build a trusting, stable and harmonious doctor-patient relationship. These are all important variables in the advancement of the Chinese health reform system.

### The Chinese CHCs are involved in delivering six main functions [2]

(1) Disease prevention and control: in disaster situations, the main task of the CHCs is to implement epidemic prevention measures effectively, while in more normal times it is to promote prevention among the residents. By following the sector's instructions on epidemic prevention, the CHC has to report carefully on the coverage of expanded planned immunizations (EPI) and the delivery of routine immunizations to children within their communities, such as carrying out community vaccine immunizations, investigating epidemics and contagious diseases, and other preventive procedures.

(2) Health care services: these include health care surveys of family members, children, women, the elderly and the disabled.

(3) Health education: by promoting geriatric and women's health education, and extending such education to students in schools around the country. In supporting the latter idea, the notion of a health-promoting school is relatively new. "The health promoting school aims at achieving healthy lifestyles for the total school population by developing supportive environments conducive to the promotion of health. It offers opportunities for, and requires commitments to, the provision of a safe and health-enhancing social and physical environment" (World Health Organization, 1993). Also, health education is essential in providing residents, those located around the CHC, with general medical and disease prevention information and treatment consultations, so that residents' concepts of care and health investment are promoted

(4) Family planning: the task here is to implement a nationwide family planning policy. This is performed by education and counseling about family planning.

(5) Medical treatment service: the main work is to consult, diagnose and treat residents/dwellers for common, frequently-occurring and chronic diseases.

(6) Community rehabilitation: this involves setting up family sick-beds, providing rehabilitation treatment and providing technical instructions for those who are considered disabled out-patients or those suffering from chronic diseases. However, owing to the scarcity of rehabilitation equipment and facilities, the community rehabilitation business is being dominated totally by TCM: acupuncture, moxibustion and massage. Thus, patients' needs can hardly be met.

After 1949, the 'socialist medical cooperation centers (SMCCs)' were established in urban and rural areas, and the health care personnel who served in these centers or paid house visits were known as 'bare-foot doctors'. Notwithstanding the ability of these centers to cover and deliver medical care to large areas, the bare-foot doctors' technical skills became out of date. However, with the country's economic development, the government started to lose control over the SMCCs because of decreased state funding, and the centers were forced to become individually owned or abandoned, which led to the disappearance of the bare-foot doctors. Between the gradual disappearance of the SMCCs and bare-foot doctors and the establishment of the CHC system, there was a transitional period of two decades (1978–1998) during which the rural areas suffered a severe lack of medical facilities; there were no private clinics. At the beginning of 1998, the government started to establish the CHC system, and in some locations the erstwhile SMCCs became clinics owned by individuals. Later, the central government started to fund these private clinics and demanded that they put their facilities and services at the disposal of their communities, by taking the role of CHCs and fulfilling their six main functions [3]. However, before the Chinese economic reform policy was implemented, the CHCs were supported financially by the government and they delivered their services free of charge to all residents. By 2004, the Ministry of Health implemented a law known as the 'Medical Security System' targeting the peasants. According to this law, the central government funded the system with 10 RMB, the local government with 10 RMB, and each individual also contributed 10 RMB. By this method, the rural areas were able to establish a CHC in each community and peasants were obliged to obtain medical services from these centers, reducing the heavy patient load on hospitals, and maintain the financing of the CHCs. As the medical industry became channeled into market-oriented business, the government's investment in the CHCs gradually started to diminish, and this led to shrinkage of the CHC services, and some CHCs were even demolished or sold to individuals. However, the number of CHCs controlled by hospitals varies depending on the development of each city. In China, the CHCs are founded by different organizations or the private sector. Some are founded by the government, some by hospitals and others are set up by the private sector. For example, in Shanghai there are a total of 86 CHCs all founded by the government but are managed by hospitals. In Guangzhou, there are 106 CHCs, but 80 of them were established and managed by hospitals, and the other 25 CHCs are set up by the neighborhoods or private people. The CHCs' management is controlled by hospitals, which is a plus for these health institutions where they are able to attract many patients through different channels, and increasing their earnings. However, the commercial aspect towards managing the CHCs by hospitals is not in the benefit of these centers because hospitals are targeting and concentrating on attracting quantity rather emphasizing on delivering a quality service. Hence, the Guangzhou government has decided to permit all CHCs of becoming independent of hospitals' control, and in the future all the CHCs in Guangzhou will be set up and managed by the local governments and neighborhoods. As for Xiamen, there are 62 CHCs, and 50 of them are set up by the government. In Fuzhou, there are 85 CHCs and 60 are founded by hospitals. In Hangzhou, there are 95 CHCs and 60 are founded by hospitals. In Zhuhai, there are 80 CHCs and 60 are founded by the private sector. So, the local governments in these cities do not support a lot the CHCs with funds but rather these centers have to be run according to the governments' medical service rules.

In the last couple of years, people have started to complain about the government's health reform policy. For example, in 2005, the authoritative research institution in China – the Development Research Center of State Council P.R. China – released results indicating that the Chinese health reform program was not successful and failed overall to deliver an efficient and cost-effective health care service to its population. Therefore, the objective of the present study is to discuss the utilization of CHCs, compare them with local hospitals, and determine whether they can deliver appropriate basic medical and public health services [4,6].

## Methods

The study was approved by Peking University Health Science Center Institutional Reviewing Board (NO: IRB00001052-T1)

All data were collected from CHCs and local hospitals with the assistance of the local health bureaux and were analyzed. In collaboration with members of the community, an initial quantitative assessment was performed to evaluate five medical resource variables: "Average professional income per staff"; "Medical income per 100 Yuan fixed assets"; "Level of person-time charge for outpatient service"; "Proportion of administrative expenditure to total expenditure"; "Average outpatients per staff per day" (Table 1). The three hospitals and three CHCs were chosen randomly. The data collected from the local health bureaux about these medical facilities differed widely in the types of services being sought and the number of patients seeking medical treatment (Table 3). The median average ages of those attending hospitals (both sexes) were between 12 to 63 years and those attending the CHCs were between 26 to 68 years (Table 4). The number of patients attending hospitals was 264 times the number attending CHCs. Furthermore, all hospital departments had a larger turnover of patients than CHC equivalents. The number and variety of departments at the CHCs is limited owing to the scarcity of resources. Also, a household survey was implemented within the community to ascertain residents' knowledge about CHCs (Table 2).

The initial quantitative assessment design was applied to six cities chosen from regions of South-East China – Guangzhou, Foshan, Shenzhen, Fuzhou, Xiamen and Hangzhou. Three hospitals (1 medium-large and two large) and three CHCs were chosen randomly "by lots", and data samples were collected from the local health bureaux. We have emphasized hospital size in order to indicate patient turnover in the outpatient departments per number of staff per day and the number of patients attending according to their needs, which vary from one hospital to another. The 3 CHCs, chosen randomly from three cities, were almost identical in the size of their facilities. Also, we investigated the utilization of health resources in each city and the standard of living in each community. All data were processed using SPSS software. Analytical methods mainly included descriptive analysis, paired T-tests, one-way ANOVA, etc. (Table 1).

We also conducted a household survey in the six cities in order to ascertain residents' knowledge about the CHCs' six functions. A total of 3000 questionnaires were distributed, 500 in each city. Of these, 2563 were returned and found valid. The remaining 437 questionnaires were invalid; 148 were returned but not fully answered, 232 were returned blank and 57 were lost. The response rate was 85.45%. The following questions were analyzed (Table 2).

## Results

Under the items "medical income per 100 Yuan fixed assets" and "proportion of administrative to total expenditure" (Table 1), there was no obvious difference between the local hospitals and the CHCs. In the three items "average professional income per staff", "level of person-time charge for outpatient service" and "average outpatients per staff", the utilization of health resources was higher in local hospitals than in the community medical health institutions. Overall, the utilization in large and medium-large local hospitals is generally higher than in the CHCs. Residents visit the local hospitals no matter whether their diseases are acute or chronic because people trust hospitals. This has led to an influx of too many people into local hospitals, which was described by the Chinese Ministry of Health as "difficult to see a doctor" and "expensive to see a doctor". The CHCs have difficulty in winning the trust of the local residents owing to the scarcity of medical resources such as lack of funds, absence of newest medical technology (diagnostic equipment could rarely be seen in these centers), and few professional and qualified medical staff (especially in the rural areas). For these same reasons, it is difficult for other community health care tasks to be carried out smoothly. In general, medical officers working in the CHCs are not qualified enough to gain the residents' trust because they are not always up to date on the latest medical information. Furthermore, many physicians working in the CHCs either have not received a bachelor degree in medicine, that is, they are medical technicians, or could not secure hospitals jobs owing to poor professional quality and turned to work in a CHC, or are specialized physicians but unable to deliver a primary care service. Furthermore, it is important to elaborate on topic concerning the medical staff working in the CHCs, about their qualification and their professional quality, which has been detected widely around the CHCs and can affect the deliverance of good or poor quality service. Since hospitals cannot dismiss their employees, hence, they displace them to the CHCs. Those who are displaced are either because they showed low quality of professionalism or because there isn't enough space to accommodate them anymore. Thus, the displaced employees work temporarily-for a short period of time until they find a suitable position in other hospitals-causing some setbacks in delivering an efficient service due to the continuous change in the staff most of the time. So, the reason behind the low quality of service in the CHCs is related to both low quality of clinicians and continuous change in the staff. There are no specific data on this issue either from the Ministry of Health or from China Statistical Bureaux. Thus, future studies are needed to explore furthermore on this issue.

Table 2 shows that the percentage of residents who have knowledge about the medical treatment performance service of the CHCs falls in the highest percentile (98.6%), and the second best-known function is prevention and control. The awareness rate of "Community rehabilitation" is 42.2% because most residents consider community rehabilitation to be part of the medical treatment function. However, residents have a low rate of awareness about the health-care service function and health education, and most responders believe that both the latter functions should be implemented by health administrators. Furthermore, residents have the same understanding about the importance of the five functions. All residents hope that the CHCs can be well constructed and effectively managed, but at the same time they are dissatisfied with the services provided by the CHCs. Satisfaction rates all fall under 70% except for "Health education", which is 75.5%. The rate for "Medical treatment service" is only 21.9%, much lower than its ratings for awareness and importance. For "Community rehabilitation" the awareness rate is the lowest (18.5%). This is mainly because of the simple equipment and poor service available, which is unable to meet residents' requirements for a rehabilitation service.

## Discussion

Since the CHCs lack the trust and support of the community, this is a real problem that needs to be solved. One has to acknowledge that deep-rooted health problems can only be solved by the people themselves in collaboration with central and local government. More emphasis should be put on the community where each neighborhood could take on a role in promoting and improving their own health care according to their own needs; an example could be taken from the U.K. model where a number of policy documents were adopted [7,8]. The data analysis indicates that the utilization of local hospitals is very much higher than that of CHCs. This is due to the high efficiency of fiscal government departments in allocating more funds to local hospitals than to CHCs. A switch of government input into CHCs might help to ensure social health equity and efficiency in utilizing health resources.

Reforming the performance of the CHCs during the last five years has brought greater convenience to local residents and helped to serve them at different levels in medical treatment, prevention, health care, recovery, health education and family planning. Residents are accepting these services to a certain extent. However, the six main functions are not fully recognized where the CHCs' services are in reality still far from reaching their targets. They have inadequate resources, low prestige and serious lack of funding and function under difficult circumstances.

### The causes to this phenomenon mainly include [3,9,10]

(1) Imperfection of compensatory funds mechanism. The government has clearly pointed out that the CHCs should be constructed within their own street communities as part of urban planning. Every street should provide housing for its CHCs. In practice, this policy is difficult to implement because house ownership and easement are always related to individuals or the interests of certain economic sectors. Since the CHCs are not channeled in accordance with the structure of society, they are institutions without clear status and lack other financing methods; for example, social donations have not reached them. Community health service centers have no single proper and unified standard charges. Charges differ widely between a hospital and a CHC. Sufferers from common diseases can obtain the service they require at the CHCs, but prefer to be treated at large hospitals. Furthermore, the double-way referral means that patients can be transferred from a CHC to a hospital, but not from a hospital to a CHC, even those with mild diseases. Hence, owing to a lack of economic compensation from the government, the CHCs are stressing treatment rather than concentrating on prevention, and this situation is widespread among the community health centers. Moreover, CHCs' expenditure is exceeding income, and they can hardly keep running on this basis [1].

(2) General medical education obviously cannot meet the residents' high demands and expectations. Almost all community doctors come from various types of hospitals with different specialties but lack primary care experience. Since they are trained only for several months in general practice, their specialist field is considered an obstacle in their performance in the CHCs, and sometimes several specialist doctors need to work together on one patient's disease. The CHCs need qualified and certified general practitioners who are considered the "curbstone general practitioners" in general medical practice.

(3) Different levels of society have different impressions of CHCs. The recognition of government leaders for these centers is not profound. Also, most residents regard the CHCs as small medical institutions that offer treatment services on the doorstep, but they do not fully understand the CHCs' six main functions. Because residents' health awareness remains weak, they do not realize the importance of the disease prevention and health care functions. When medical personnel pay a home visit in order to perform a health promotion or complete a family health record, a misconception frequently arises; residents think that the CHC personnel might charge for the service or "though no charge now, charge in the future". So they refuse the service that has been offered to them, and this hinders the CHCs' work. Hence, health leaders should have a better recognition and understanding of these issues, and health promotion should concentrate more on education targeting the service users.

(4) The CHCs lack experience in the operation and management fields. The institutions' efficiency and performance are affected by heavy bureaucracy: there is a lack of research that could be utilized to tackle problems; an absence of effective assessment methods or system for evaluating management; no institutional incentives such as a staff performance reward or punishment system; and no effective supervisory management system throughout the operation.

The government must exert more effort by performing more serious research into residents' health care needs. The central and local governments should take advantage of the information available about peoples' needs and demands in order to develop the service areas by expanding the service connotations plan and making the CHCs develop healthily and continuously.

### Targeting these problems, we propose several suggestions for the CHCs [11,12]

One should take a further step towards perfecting the CHCs' economic compensation methods. Of the CHCs' six main functions, only medical treatment can bring an economic yield. The other five functions have economic yields that are not obvious and they have "welfare" features. The national government proposed that the CHCs' economic compensation channels should include government participation in subsidizing the costs for residents attending the CHCs; direction of part of hospitals' pharmaceutical income towards financing the CHCs; permitting local residents, those holding the medical insurance, to be treated at the CHCs; etc. In future, the government should pay more attention to the CHCs' role when it is considering health cost investment. Currently, the service sectors and the types of treatment within the CHCs differ from what hospitals offer. They have no standardized or a unified charge. To make up for the shortages of funds, the government should exert more effort to formulate unified prices. Each standardized charge should be suitable for the local residents' level of income, economic affordability and medical insurance reforms. The CHCs should be eligible for listing in the urban resident's basic medical insurance expense account, which would help to build a "double-way referral mechanism", i.e., patients with serious diseases should be transferred from the community medical centers to hospitals, and patients with mild diseases should be transferred from hospitals to the CHCs. This will reduce medical expenses as a whole.

(1) Build and improve effective management systems for CHCs. Following the basic principle of "government leads CHC, street institution builds stage for CHC, and CHC serves residents", the construction of a management system should channel the CHCs under the government's control. Meanwhile, sufficient consideration should be given to both variables: the industry/profession and the region. It is necessary that a norm for operational management be formulated, specific patterns be followed, and necessary functions be implemented by the community health service, such as an evaluation system and a community health service assessment index. By adopting these two evaluation scales, the CHCs will be more standardized and normalized.

(2) Strengthening the construction of the CHCs' professional staff. Currently, according to the government's proposals and requirements, every CHC should serve 2000 to 4000 people and should have a general practitioner (GP). There is an urgent need to implement a scheme to train high qualified and accomplished GPs. Another important issue is that, when we select and allocate community health service personnel, we should pay more attention to their formal schooling, major, knowledge, age, professional job title, etc. Those with current low professional job titles and low formal schooling should be replaced by highly qualified staff. Since general medical education is considered the core of medicine, post training is considered essential. Current and future community health service workers should receive standard post training and should be licensed to work after they have passed the assessment tests. By adopting this set of standards, the quality of the general service will gradually be improved. We should emphasize the work performance reward and punishment system, linking performance with placement and job titles with promotion, in order to stimulate initiatives among the medical care workers serving the communities.

(3) Implement promotion and continuous medical education at different levels. With the new health service reforms, the CHCs require the wholehearted support of society as a whole and broad participation from residents living within their communities. Lobbying and promoting widely will push the leaders to change their way of thinking and strengthen their goals towards the construction of CHCs. The central and local health authorities should institute further promotion and implement further continuous education for the medical staff. Also, when evaluating the performance of CHC medical staff, their attitude towards patients should take "biological-psychological-social behavior" into consideration [3]. The health providers should not only observe the patient as a single individual but rather as part of, and influenced by, society. Chinese health care providers must change their way of thinking in treating the individual by applying psycho-somatic therapy first, and this may be followed by treating the disease if necessary. Thus, the main function of the CHCs must change from treatment to prevention and health care.

## Conclusion

Efficient essential health care should be available to everyone. The local hospitals and the CHCs are considered the main care providers for local residents. It is important to put the six functions of the CHCs into effect and build a new and safe community health care network [1,5].

However, there are some problems in realizing the functions of the CHCs:

(1) CHC services are far from understood. The rates of awareness about Community rehabilitation, Health care service and Health education are 42.2%, 35.3%, 31.8%, respectively. These functions are not fully realized or completely utilized.

(2) At present, the CHCs' main function is "medical treatment service", and the awareness rate of "Medical treatment service" is the highest (98.6%). However, the rate of "Satisfaction about the item" is the lowest (21.9%).

(3) As a whole, the utilization of medium-large to large hospitals is generally higher than that of CHCs. Residents prefer to attend local hospitals whether their diseases are serious or not. This has led many people to complain about the service, described by the Chinese Ministry of Health as "difficult to see doctor" and "expensive to see doctor" in some hospitals [2,5,6].

To sum up: we need to increase the promotion of health education towards residents; try to change the residents' perception and recognition of health care; try to understand the concept of health consumption by making the CHCs' six service functions suitable for people who have high expectations and demands for appropriate health care; and make the community health service function better.

## Competing interests

The author(s) declare that they have no competing interests.

## Authors' contributions

*Corresponding authors

*Prof PX designed the study, carried out the field work and wrote the initial draft.

*Dr. HD analyzed the data and guided and supervised the design and participated in writing and finalizing the paper. Both authors read and approved the final manuscript. 

WX performed field work, collected the data and participated in its statistical analysis. 

ZH performed field work, collected the data and participated in its statistical analysis.

## Pre-publication history

The pre-publication history for this paper can be accessed here:



## Figures and Tables

**Table 1:** Analysis of Utilization of Health Resources: Comparison between Hospitals and CHCs

Item	Type	Mean ± sd (RMB Yuan)	*t*-value	P value
Average professional Income per staff	hospital	164287.1 ± 872.3	8.50	0.001
	CHC	52292.0 ± 479.4		
Medical income per 100 Yuan fixed assets	hospital	66.4 ± 8.65	0.72	0.479
	CHC	54.4 ± 4.57		
Level of person-time charge for outpatient service	hospital	98.9 ± 10.3	12.26	0.001
	CHC	25.5 ± 3.45		
Proportion of administrative to total expenditure	hospital	18.5 ± 5.38	0.89	0.523
	CHC	15.6 ± 4.63		
Average outpatients per staff	hospital	1095.3 ± 67.8	4.93	0.001
	CHC	343.5 ± 15.7		

**Table 2:** Residents' Knowledge about CHC Functions

Items	Just know it (%)	Think it is important (%)	Satisfaction about the item (%)
Prevention and control	78.6	99.2	66.1
Healthcare service	35.3	72.8	62.6
Health education	31.8	76.1	75.5
Medical treatment service	98.6	83.6	21.9
Family planning	75.6	70.9	63.2
Community rehabilitation	42.2	67.9	18.5

**Table 3:** Data showing patients' needs in seeking medical treatment in medium-large to large hospitals per day

Dept	No of patients	Age (yrs)	Sex (M/F)	Chr D (M/F)	Act D (M/F)
	Total px's/day 8980				
Endo	1380	11–48	835/545	502/257	333/288
CVD	2010	35–73	1123/887	857/412	266/475
RTD	1322	32–81	963/359	737/232	226/127
GITD	848	23–56	366/482	113/121	253/367
Inf D	760	5–64	418/348	241/213	177/135
STD	830	26–58	527/303	141/206	386/97
Surg	129	4–67	41/88	18/37	23/51
Derm	640	15–79	378/262	223/149	155/113
Renal D	450	12–63	168/282	132/154	36/128
Peds	317	1m-15	123/194	39/54	69/140
Ob/Gyn	294	23–48	0/244	0/183	0/67

Endo = Endocronology; CVD = Cardiovascular Disease; RTD = Respiratory Tract Disease; GITD = Gastroenterology Disease; Inf D = Infectious Disease; STD = Sexual Transmitted Disease; Surg = Surgery; Derm = Dermatology; Renal D = Renal Disease; Peds = Pediatrics; OB/GYN = Obstetrics & Gynecology; Chr = Chronic; Act = Acute; D = Disease; M = Male; F = Female; px's = patients

**Table 4:** Data collected from three CHCs indicating the number of patients seeking medical treatment

Dept	No of patients	Age (yrs)	Sex (M/F)	Chr D (M/F)	Act D (M/F)
	Total px's/day 34				
CVD	11	38–63	7/4	5/2	2/2
RTD	7	41–68	6/1	4/1	2/0
GITD	5	25–76	3/2	2/2	3/0
Inf D	7	26–49	4/3	2/1	5/2
Derm	4	15–71	1/3	1/1	3/2
